# Effects of the Probiotic *Enterococcus faecium* and Pathogenic *Escherichia coli* Strains in a Pig and Human Epithelial Intestinal Cell Model

**DOI:** 10.1155/2015/235184

**Published:** 2015-03-26

**Authors:** Ulrike Lodemann, Julia Strahlendorf, Peter Schierack, Shanti Klingspor, Jörg R. Aschenbach, Holger Martens

**Affiliations:** ^1^Institute of Veterinary Physiology, Faculty of Veterinary Medicine, Freie Universität Berlin, 14163 Berlin, Germany; ^2^Institute of Microbiology and Epizootics, Faculty of Veterinary Medicine, Freie Universität Berlin, 10115 Berlin, Germany; ^3^Faculty of Natural Sciences, University of Applied Sciences, 01968 Senftenberg, Germany

## Abstract

The aim of this study has been to elucidate the effect of the probiotic *Enterococcus faecium* NCIMB 10415 on epithelial integrity in intestinal epithelial cells and whether pre- and coincubation with this strain can reproducibly prevent damage induced by enterotoxigenic (ETEC) and enteropathogenic *Escherichia coli* (EPEC). Porcine (IPEC-J2) and human (Caco-2) intestinal epithelial cells were incubated with bacterial strains and epithelial integrity was assessed by measuring transepithelial electrical resistance (TEER) and mannitol flux rates. *E. faecium* alone increased TEER of Caco-2 cells without affecting mannitol fluxes whereas the *E. coli* strains decreased TEER and concomitantly increased mannitol flux rates in both cell lines. Preincubation with *E. faecium* had no effect on the TEER decrease induced by *E. coli* in preliminary experiments. However, in a second set of experiments using a slightly different protocol, *E. faecium* ameliorated the TEER decrease induced by ETEC at 4 h in IPEC-J2 and at 2, 4, and 6 h in Caco-2 cells. We conclude that *E. faecium* positively affected epithelial integrity in monoinfected Caco-2 cells and could ameliorate the damage on TEER induced by an ETEC strain. Reproducibility of the results is, however, limited when experiments are performed with living bacteria over longer periods.

## 1. Introduction

Following the ban of antibiotic growth promoters in farm animals in the European Union in 2006, the search for alternative growth and health promoters has been intensified. Among such promoters, probiotics seem to be a suitable feed additive promoting health and performance parameters of piglets [1–3]. However, research with regard to their application in farm animals is still fragmentary and our present knowledge concerning the underlying mechanisms of the effects of probiotics is limited.

*Enterococcus faecium* NCIMB 10415 (*E. faecium*) is used as a probiotic supplement in farm animals. It has shown positive effects on diarrhoea incidence [4, 5] and daily weight gain [5] in pigs. Furthermore, effects on immunological parameters in the gastrointestinal tract [6–8] and effects on transport properties in the pig jejunum [9] have been observed.

The mechanisms of these effects are still not well understood. For a better understanding of the interaction between intestinal cells and probiotics, cell models can be used, as done with human probiotic preparations [10–12].

Therefore, we have examined the effects of* E. faecium* in a model of porcine intestinal epithelial cells, namely, in IPEC-J2 cells isolated from the jejunum of a newborn piglet [13, 14]. Their usefulness as a model for studies of microbial pathogenesis in pigs has previously been assessed [15]. Up to now, these cells have been used in probiotic studies with different strains, including assessments of the adhesion capability of various probiotic strains such as* Lactobacillus plantarum*,* Enterococcus faecium* EF2019, and* Saccharomyces cerevisiae* [16–19] and of the capability of probiotics to inhibit inflammatory responses [18, 20] or their antiviral activity [21].

In the present study, we have focused on the barrier function of the epithelial cells. We have compared a human cell line, namely, Caco-2, an established* in vitro* model of small intestinal mucosa, and a porcine cell culture model, namely, IPEC-J2, with regard to their response to incubation with probiotic* E. faecium* and pathogenic* Escherichia coli* strains. We have addressed the question as to whether* E. faecium* and two selected pathogenic* E. coli* strains affect the barrier function of porcine intestinal epithelial cells. Furthermore, we assumed that the effects of pathogenic strains can be modified by* E. faecium*. In the performed experiments, intestinal epithelial cells were incubated long-term with live bacteria.

## 2. Materials and Methods

### 2.1. Cells and Culture Conditions

The porcine intestinal epithelial cell line (IPEC-J2) was established from the jejunum of a newborn piglet [13, 14] and kindly provided by Professor Anthony Blikslager (North Carolina State University, USA). The IPEC-J2 cells were maintained in Dulbecco's modified Eagle medium (DMEM)/Ham's F-12 medium (1 : 1), supplemented with 5% fetal bovine serum (FBS, Biochrom, Berlin, Germany), 2.5 mmol/L L-glutamine (Biochrom, Berlin, Germany), insulin (5 *μ*g/mL), transferrin (5 *μ*g/mL), selenium (5 ng/mL) (ITS, Sigma-Aldrich Chemie GmbH, Taufkirchen, Germany), epidermal growth factor (EGF, 5 ng/mL, Biochrom, Berlin, Germany), and penicillin-streptomycin (Sigma-Aldrich Chemie GmbH, Taufkirchen, Germany). IPEC-J2 cells were passaged by trypsinization (0.15 g/L porcine trypsin, 0.06 g/L EDTA, Sigma-Aldrich Chemie GmbH, Taufkirchen, Germany). Cells were used consistently within 14 days from the seeding of passages 69–79.

Human epithelial intestinal cells from colorectal adenocarcinoma, Caco-2 (ATCC Catalog number HTB-37, ATCC, Manassas, USA) were maintained in Eagle's minimum essential medium with Earle's BSS and 2 mmol/L L-glutamine (EMEM, LGC Standards GmbH, Wesel, Germany) modified by ATCC to contain 1.0 mmol/L sodium pyruvate, 0.1 mmol/L nonessential amino acids, 1.5 g/L sodium bicarbonate, and supplemented with 20% FBS and penicillin-streptomycin. Cells were studied between passages 33 and 46 and subcultured every 4-5 days, after trypsin treatment (2.5 g/L porcine trypsin and 0.2 g/L EDTA, Sigma-Aldrich Chemie GmbH, Taufkirchen, Germany).

Both cell lines were grown at 37°C in a humidified atmosphere of 5% CO_2_. Cell cultures were routinely tested and found to be free of* mycoplasma* contamination. On the day prior to experiments, the cells were fed with serum- and antibiotic-free medium.

### 2.2. Transepithelial Electrical Resistance (TEER) Measurements

For TEER, mannitol, and pH measurements, the cells were seeded on clear polyester membrane cell culture inserts (Snapwell, 12 mm diameter, 1.12 cm^2^ area, 0.4 *μ*m pore size; Corning B.V., Schiphol-Rijk, Netherlands). The membranes were coated with rat tail collagen type I (Serva Electrophoresis GmbH, Heidelberg, Germany) for IPEC-J2 cells. Cells were seeded at a density of 10^5^ cells/1.12 cm^2^ and were allowed to differentiate for 14 days (IPEC-J2) or 21 days (Caco-2). TEER measurements were performed by using a Millicell-ERS (Electrical Resistance System; Millipore GmbH, Schwalbach, Germany). TEER values were corrected for the resistance of blank filters and for the membrane area. Measurements were started after the cell monolayers had reached confluency.

### 2.3. Bacterial Strains

Cells were incubated with bacteria from the (1) probiotic strain* Enterococcus faecium* NCIMB 10415 (cultivated from Cylactin; Cerbios-Pharma, Barbengo, Switzerland, and provided by David Taras, Institute of Animal Nutrition, Berlin, Germany), (2) enterotoxigenic* E. coli* IMT4818 (ETEC, isolated from a two week-old piglet with enteritis, O149:K91:K88 (F4) and found to be positive for the presence of virulence genes est-1a, est-2 (genes coding for heat stable enterotoxins I and II), and elt-1a/b (gene coding for heat labile enterotoxin I) by polymerase chain reaction (PCR)), or (3) human enteropathogenic* E. coli* E2348/69 (EPEC, serotype O127:H6 and positive for eae (*E. coli* attaching-effacing) gene).

*E. faecium *NCIMB 10415 was grown in brain-heart infusion (BHI) broth (OXOID GmbH, Wesel, Germany) and the ETEC and EPEC strains were grown in LB medium containing 10 g/L tryptone, 5 g/L yeast extract, and 10 g/L NaCl, at a pH of 7.0. Tryptone and the yeast extract were from OXOID.

After overnight incubation at 37°C, subcultures of bacteria grown for ~3 h until midlog phase were centrifuged and washed twice in phosphate-buffered saline (PBS, Biochrom, Berlin, Germany). Bacterial cells were then resuspended in antibiotic- and serum-free IPEC-J2 or Caco-2 cell culture medium at a concentration of ~10^8^ colony-forming units (CFU)/mL. From this solution, aliquots were added to the apical compartment of the cell culture inserts, which contains 0.5 mL media, to reach the concentrations indicated below.

Bacterial concentration was determined by measuring the optical density and confirmed by serial dilution followed by determining viable counts on agar plates (Columbia blood agar, PC agar for all strains, Endo agar for* E. coli* strains, and Citrate Azide Tween Carbonate Agar for* E. faecium*) in preliminary experiments. Cells were infected with 10^6^, 10^7^ and, for the* E. faecium* strain, also with 5 × 10^6^ bacteria per cell culture insert (1.12 cm^2^), corresponding to a multiplicity of infection (MOI) of 10, 50, and 100 bacteria, respectively, per seeded cell. When the cells were infected with* E. faecium* and either the ETEC or the EPEC together, cells were preincubated with* E. faecium* for 3 h in the first set of experiments and for 2 h in the second set of experiments and, after that, the pathogenic* E. coli* strains were added. The cells were in contact with the* E. coli* for the same duration as in the monoincubation monolayers with either ETEC or EPEC. This setup will be called “coincubation” in the following and the incubation time will be given as the time that the cells were incubated with the pathogenic* E. coli* strains.

### 2.4. Measurements of ^3^H-Mannitol Fluxes

Flux rates of mannitol were measured by using D-[1-3H]-mannitol (PerkinElmer Life Sciences, Rodgau-Jügesheim, Germany). The isotope (0.3 *μ*Ci) was added to the apical side of the cell monolayers, and the cells were incubated for 30 min to allow equilibration of the isotope. Fluxes were calculated from the rate of appearance of tracer on the serosal side of the cell monolayer within 60 min (three fluxes of 20 min). D-[1-3H]-Mannitol was assayed by using a well-type crystal *β*-counter (LKB Wallace-PerkinElmer, Überlingen, Germany).

### 2.5. pH Measurements

The pH of cell culture media at the apical side of the cell monolayers was measured with an InLab Surface electrode (Mettler Toledo Online GmbH, Nänikon, Switzerland) connected to a pH meter (inoLab pH 720, Wissenschaftlich-Technische Werkstätten GmbH, Weilheim, Germany).

### 2.6. Statistical Analysis

Statistical evaluations were carried out by means of the PASW Statistics program for Windows, version 18 (Jandel, Chicago, IL, USA). Graphs were plotted with Excel 2010. Unless otherwise stated, results are given as mean ± standard error of the mean. The number of cell monolayers that entered the statistical evaluation is indicated in the relevant tables and figure legends. Results were considered to be significant at *P* ≤ 0.05.

To assess the effect of the various numbers of bacteria (“dose”) on TEER, a one-way analysis of variance with the fixed factor “dose” (0, 10^6^, 5 × 10^6^, 10^7^/1.12 cm^2^ or 0, 10^6^, 10^7^/1.12 cm^2^), was conducted per time point (h) with a post hoc Scheffe test. For effects of incubation with* E. faecium* on TEER, a one-way analysis of variance with the fixed factor “treatment” (control,* E. faecium*) was conducted per time point. For effects of bacterial incubation and preincubation with* E. faecium* on TEER, a one-way analysis of variance with the fixed factor “treatment” (control:* E. faecium*, ETEC, and* E. faecium* + ETEC; control:* E. faecium*, EPEC, and* E. faecium* + EPEC) was conducted per time point with a post hoc Scheffe or LSD (least significant difference) test.

## 3. Results

### 3.1. Determination of the Incubation Conditions

TEER reached values of ~5000 Ohm × cm^2^ in confluent IPEC-J2 cell monolayers after 14 days in culture and ~550 Ohm × cm^2^ in Caco-2 cells after 21 days. Next, we optimized the incubation conditions for the infection model by testing various dosages of the* E. faecium* (10^6^, 5 × 10^6^, and 10^7^ CFU/cell culture inserts) and the two pathogenic* E. coli* strains (10^6^ and 10^7^ CFU/cell culture inserts).

**Figure 1:**
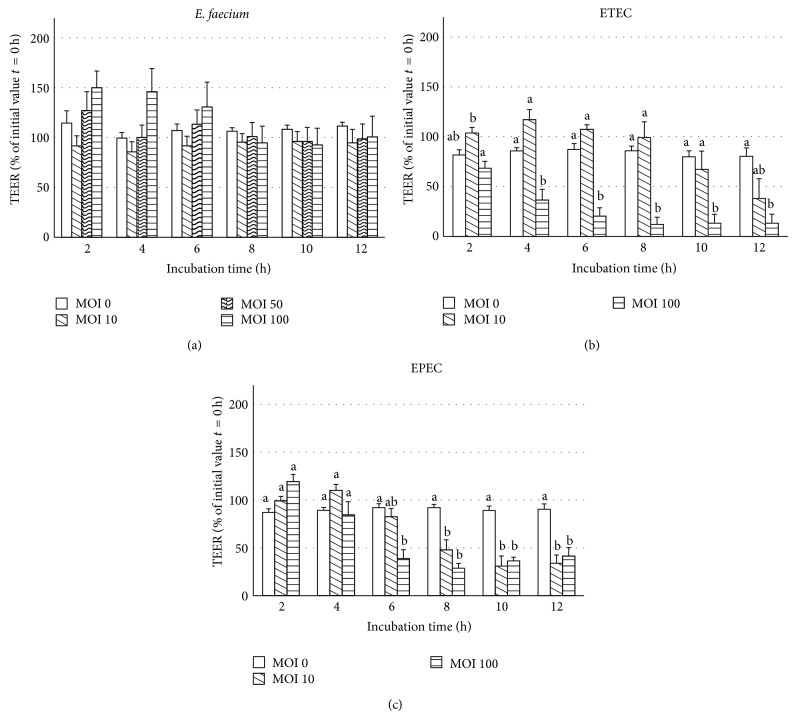
TEER values (% of initial value *t* = 0 h) of IPEC-J2 cells during 12 h of incubation with various doses of (a)* E. faecium*, (b) ETEC, or (c) EPEC (MOI = multiplicity of infection per seeded cell) on Snapwell collagenized polyester membranes (mean ± SEM), *n* = 6–11 cell culture inserts. Different letters indicate significant differences between treatment groups per time point (*P* ≤ 0.05).

Changes of TEER were measured over 12 h in IPEC-J2 cells after mucosal addition of bacterial strains (Figure 1). When the cell monolayers were exposed to increasing doses of* E. faecium*, the TEER values showed no significant differences (Figure 1(a)). In contrast, in IPEC-J2 cell monolayers incubated with the ETEC strain, the highest infection dose induced a significant decrease in TEER beginning at 4 h and reaching values of ~20% of the initial value after 6 h (Figure 1(b)). When the IPEC-J2 cells were incubated with EPEC, the TEER was significantly decreased compared with the control from 6 or 8 h onwards at the highest and the lower infection doses, respectively (Figure 1(c)).

**Figure 2:**
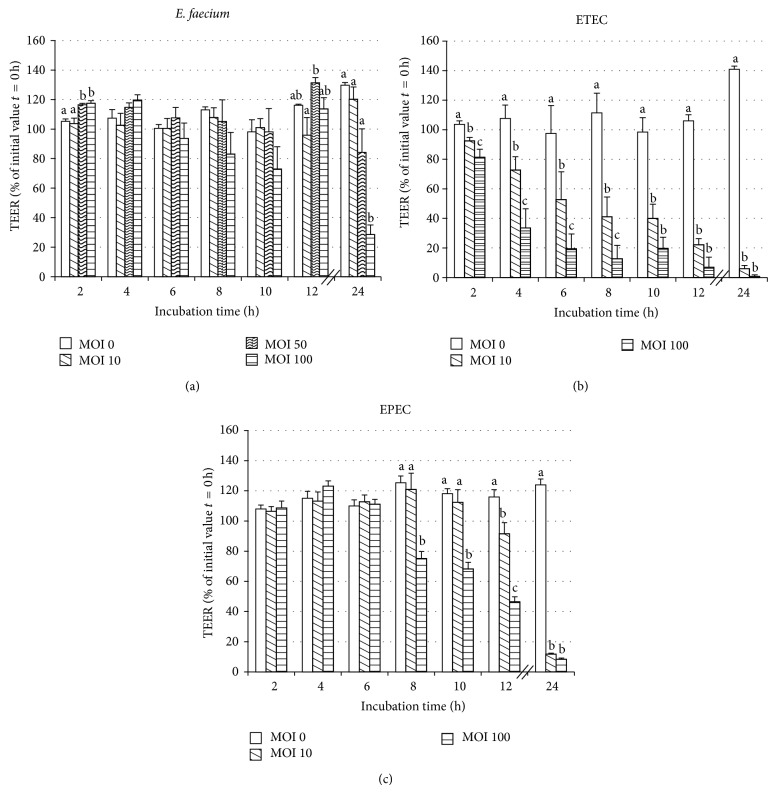
TEER values (% of initial value *t* = 0 h) of Caco-2 cells during 24 h after treatment with various doses of (a)* E. faecium* (b) ETEC, or (c) EPEC (MOI = multiplicity of infection per seeded cell) on Snapwell collagenized polyester membranes (mean ± SEM), *n* = 5–9 cell culture inserts. Different letters indicate significant differences between treatment groups per time point (*P* ≤ 0.05).

In Caco-2 cells, the highest dose of* E. faecium* induced a decrease in TEER after 24 h (Figure 2(a)).

When the Caco-2 cells were incubated with ETEC, the TEER decreased in a concentration-dependent manner starting already at 2 h (Figure 2(b)). In contrast, when infected with EPEC, the TEER did not significantly differ from the control group until 8 h (Figure 2(c)). Thereafter, a decrease was observed at the highest infection dose and, after 12 h, also at the lower infection dose.

In some of the experiments, mannitol flux rates were measured, as a marker of paracellular permeability, in parallel to the measurement of TEER (see Tables 1 and 2). The mannitol flux rates reflected the changes in TEER.

### 3.2. Effects of the Probiotic* E. faecium* on TEER

**Figure 3:**
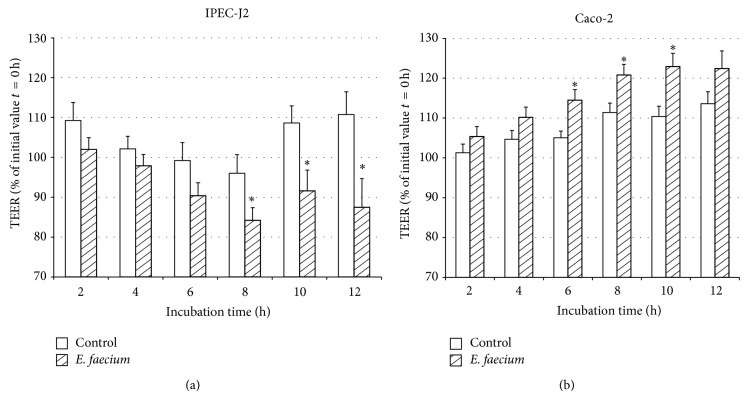
TEER values (% of initial value *t* = 0 h) of IPEC-J2 cells (a) and Caco-2 (b) cells during 12 h of incubation with control medium or* E. faecium* (10^6^ CFU per cell culture insert) (mean ± SEM), IPEC-J2: *n* = 22–39 cell culture inserts; Caco-2: *n* = 16–36 cell culture inserts. The symbol ∗ indicates a difference between* E. faecium*-incubated cells and control cells.

We chose an* E. faecium* concentration of 10^6^ bacteria/1.12 cm^2^ for all further experiments and observed an enhancing effect on TEER over 12 h in Caco-2 cell monolayers, which was significant from 6 h to 10 h  (Figure 3(b)). No such effect was observed in IPEC-J2 cell monolayers. Instead, the TEER of IPEC-J2 cell monolayers incubated with* E. faecium* significantly decreased from 8 h onwards in this experimental series (Figure 3(a)).

### 3.3. Effects of Coincubation with* E. faecium* on Pathogenic Challenge in a First Series of Experiments

As we were interested in whether the effects of a pathogenic challenge by the* E. coli* strains could be prevented by the probiotic* E. faecium*, we preincubated the epithelial cells for 3 h with* E. faecium*, after which time the pathogenic* E. coli* strains were added. In parallel, cell monolayers were monoinfected with the respective strains, the incubation lasted for 12 h (IPEC-J2) and 24 h (Caco-2), respectively. In both cell lines,* E. faecium* did not reproducibly delay the decrease in TEER induced by ETEC and EPEC (data not shown).

The pH in cell culture inserts was measured at the apical side of the cell monolayer. For IPEC-J2 cells, the pH values decreased with time and were below 6.5 after 10 h of incubation with EPEC and the* E. faecium* coincubation with ETEC and EPEC. The pH of Caco-2 cell media decreased below 7 after 10 h for the* E. coli* strains and their coincubations (data not shown).

### 3.4. Effects of Coincubation with* E. faecium* on Pathogenic Challenge in a Second Set of Experiments

**Figure 4:**
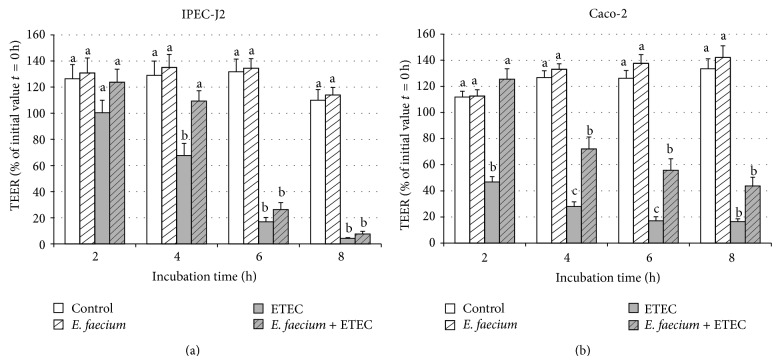
TEER values of IPEC-J2 (a) and Caco-2 (b) cells during 8 h of treatment with various bacterial strains: control without bacteria,* E. faecium*, ETEC,* E. faecium* + ETEC, incubation time adjusted to the addition of the* E. coli* strain (mean ± SEM), and *n* = 11-12 cell culture inserts per time point. Different letters indicate significant differences between treatment groups per time point (*P* ≤ 0.05).

Based on the results of the first set of experiments, we conducted a further set of experiments with shorter incubation times. In these experiments, only the effects of ETEC were tested, taking care to exactly match the duration of incubation of cells with the pathogenic* E. coli* in both monoinfected and coincubated cells. In these experiments, the coincubation with* E. faecium* could ameliorate the decrease in TEER induced by ETEC at 4 h in IPEC-J2 and at 2, 4, and 6 h in Caco-2 cells (see Figure 4).

## 4. Discussion

Probiotic feed supplementation is an alternative for the use of antibiotic growth promoters in farm animals that is now prohibited in the EU.* E. faecium* NCIMB 10415 is accredited as a feed supplement for piglets and has positive effects on health incidence and performance parameters [4, 5], which cannot be satisfactorily explained on the basis of our present knowledge. The possible improvement or protection of the intestinal barrier function has been discussed as one possible mechanism of probiotic action [11, 12]. The present study has been undertaken to determine the validity of these arguments. The effects of* E. faecium* and two selected pathogenic bacterial* E. coli* strains on TEER of IPEC-J2 (pig) and Caco-2 (human) cell monolayers have been investigated. Of particular interest was the potential beneficial effect of* E. faecium* on the barrier integrity of the IPEC-J2 cell line, because studies regarding the complete or partial abrogation of the impairing effects of pathogenic* E. coli* by probiotics have mostly been conducted with human cell lines and probiotics used in human nutrition [22–26].

### 4.1. Experimental Model

We used live bacteria in our study, because various cell structures or secreted factors, such as bacterial DNA, secreted substances, and cell wall components, might be responsible for any effects induced. The infection model proved difficult to handle because the time course of the change in TEER after addition of bacteria differed between experiments conducted on different days. The latter occurred despite due care to have a highly standardized procedure and may point to the fact that even minor differences in cell passages and batches of bacterial culture may have huge impacts on the experimental outcome. An overall statistical evaluation of the results of multiple independent experiments was therefore difficult. In addition, we used live bacteria over a long period of time, during which they multiplied in the cell culture wells. This had effects on the pH of the cell culture media which declined and hence the data from later time points could have been influenced by the change in pH. We therefore reduced the duration of the incubation time when we tested the probiotic effect on changes induced by ETEC in a second set of coculture experiments.

### 4.2. Effects of* E. faecium* Per Se on TEER

TEER is measured as a parameter to assess the variation of the integrity and permeability of the epithelial barrier [27–29] and is given by the cellular resistance, *R*_*c*_, and the shunt resistance, *R*_*s*_, which operate in parallel. Any change of TEER can be caused by changes of *R*_*c*_, *R*_*s*_, or both.

The* E. faecium* strain had either no effect on TEER or resulted in a decrease in the TEER in IPEC-J2 cells from 8 h of incubation onwards. In Caco-2 cells, the TEER was increased by* E. faecium* at a concentration of 10^6^/1.12 cm^2^. The latter findings are in general agreement with results from other probiotics (*Bifidobacterium infantis*,* E. coli Nissle* 1917,* Lactobacillus acidophilus*,* Streptococcus thermophilus*,* VSL#3*,* L. rhamnosus*, and* Saccharomyces boulardii*) and cell lines (T84, HT29/cl.19A, and Caco-2) [30–34]. The application of probiotics alone, without a pathophysiological challenge, in* in vitro* studies with intestinal epithelia has either an enhancing effect [30–32] or no effect on TEER [26, 33, 34], with the effect being dose- and time-dependent [31, 35, 36].

### 4.3. Effect of* E. faecium* during Pathogenic Challenge

We furthermore hypothesised that* E. faecium* could prevent decreases in TEER in a pathophysiological challenge. Therefore, we chose two pathogenic* E. coli* strains, one isolated from pig and the other from human, that decreased TEER in a time- and dose-dependent manner specific to the bacteria and their mode of action and cell lines used. This decreasing effect on TEER confirmed results of similar experiments with different cell lines, bacteria, or infection doses. A TEER decrease by* E. coli* O149K91 (F4 (K88ac) (ETEC) with heat labile (LT+) and heat stable (STb+) enterotoxins) in IPEC-J2 cells has also been observed by Geens and Niewold [37] at MOI of 10 : 1 (to 2% of the initial value after 4 h). Similar results have been reported for Caco-2 cells in the study of Roselli et al. [38] in which the TEER fell to about 50% after 3 h (5 × 10^7^ bacteria/1.12 cm^2^). This disruption of barrier function by ETEC might in part be attributable to lipopolysaccharide or bacterial metabolites [37].

In Caco-2 cells incubated with EPEC (EPEC O127:H6, E2348/69), the TEER fell below 50% at 4 h in the study of Anderson et al. [39]. The decrease in TEER after incubation with EPEC is a result of the disruption of tight junction integrity by two type III secreted effector molecules and a bacterial surface protein (Dean and Kenny, 2004, and Hecht, 2001). It is also possible that induction of enterocyte apoptosis contributes partly to the TEER increase after EPEC exposure. However, it has been previously shown that changes in epithelial resistance can occur independent of the induction of epithelial cell apoptosis, at least, in Caco-2 cells [40].

The effects on TEER were reflected by the changes in the mannitol flux rates as a marker for paracellular permeability. The decrease in TEER and concomitant increase in mannitol flux in response to the pathogenic* E. coli* strains commonly point to an opening of the paracellular permeation pathway and could be due to a change or delocalization of TJ or cytoskeletal proteins. Probiotic bacteria such as* L. plantarum*,* L. acidophilus*, or* L. rhamnosus* have previously been shown to prevent these effects of* E. coli* on barrier function in studies with Caco-2 or T84 cells [33, 39]. For example,* L. sobrius* DSM 16698 protected IPEC-1 cells (derived from porcine jejunum and ileum) from the disruption of TJ structure by inhibiting the delocalization of ZO-1, the reduction in the amount of occludin, the rearrangement of F-actin, and the dephosphorylation of occludin caused by an enterotoxigenic* E. coli* strain [41]. This has also been shown in animal studies* in vivo*;* for example*, one week of pretreatment with* L. plantarum* in the drinking water abolished the* E. coli*-induced increase of mannitol passage in the small intestine of rats [42].

The ETEC and EPEC strains have been used in the current study as a pathogenic challenge to examine whether the decrease observed in TEER can be prevented or reduced by the probiotic* E. faecium*. The cell lines were preincubated with the probiotic before the application of ETEC or EPEC. This design was chosen based on the observation that in most of the studies in which pathogens or other damaging agents have been employed, only pretreatment with the probiotic has produced the designated effect [22, 33]. Preincubation with* E. faecium* did not diminish the effects of the pathogenic strains ETEC and EPEC in preliminary experiments but diminished the TEER decrease induced by ETEC in the second set of experiments.

As discussed above, the decrease in TEER induced by pathogenic* E. coli* strains was different on several days and in the preliminary challenge experiments the decrease in TEER started later. Furthermore, we used different preincubation times (3 h and 2 h) with the probiotic strain before the pathogenic strain was added.

Although the main effects towards incubation with bacteria were rather similar, some differences between the two cell lines could be observed. When the cells were incubated with ETEC strain, Caco-2 cells reacted earlier with a decrease in TEER and at lower bacterial concentrations (MOI 10) than IPEC-J2 cells (Figures 1, 2, and 4). Oppositely, the decrease in TEER induced by EPEC occurred generally later than that observed after addition of ETEC, with IPEC-J2 cells reacting slightly earlier. The ETEC strain was isolated from a piglet and the EPEC strain is a human strain and it might be speculated that the IPEC-J2 cells might be more adapted to the porcine pathogen. Besides species differences, the differences between the cells might also be due to their origin (tumorous or nontumouros tissues) and might also be influenced by the cell-specific media (e.g., regarding buffering capacity). As stated by Geens and Niewold [43] IPEC-J2 may represent a better model of normal intestinal epithelial cells than transformed cell lines because they maintain their differentiated characteristics and exhibit strong similarities to primary intestinal epithelial cells.

## 5. Conclusions

The increasing application of probiotics in animal nutrition requires an investigation of the underlying mechanisms of their health-promoting effects.* E. faecium* NCIMB 10415, which has been accredited for piglet nutrition, induces an increase in TEER in human epithelial cell monolayers, but not in the porcine intestinal cell model. Pre- and coincubation with* E. faecium* could ameliorate the decrease in TEER induced by enterotoxigenic* E. coli*. However, the effects of the probiotic strain in pathogenic challenges differed in two experimental setups and for further experiments shorter incubation times should be used.

## Figures and Tables

**Table 1:** Mannitol flux rates and their corresponding TEER values in IPEC-J2 cell monolayers incubated with various bacterial strains.

	Control	Ecf	ETEC	EPEC	Sampling time
TEER (Ohm∗cm^2^)	4760 ± 1283	4744 ± 1187	3021 ± 2341	4590 ± 1263	4 h
Mannitol flux (nmol∗cm^−2^∗h^−1^)	4.4 ± 3.2	3.9 ± 3.7	6.5 ± 5.4	15.2 ± 13.8	
*n* TEER∣*n* Mannitol	14∣6	15∣7	14∣9	12∣6	
TEER (Ohm∗cm^2^)	3518 ± 1397^a^	3692 ± 1177^a^	2690 ± 2428^ab^	1115 ± 1018^b^	8 h
Mannitol flux (nmol∗cm^−2^∗h^−1^)	2.1 ± 2.6^a^	3.4 ± 2.0^a^	11 ± 12.2^ab^	26.9 ± 25.1^b^	8 h
*n* TEER∣*n* Mannitol	13∣8	14∣7	13∣4	11∣6	

Bacterial strains: IPEC-J2 cells were incubated with *E. faecium* (Ecf), ETEC, or EPEC for 4 h and 8 h (mean ± standard deviation, variance analysis, post hoc Scheffe test, *P* ≤ 0.05, and different letters indicate significant differences between incubation groups); *n* = cell culture inserts.

**Table 2:** Mannitol flux rates and their corresponding TEER values in Caco-2 cell monolayers incubated with various bacterial strains.

	Control	Ecf	ETEC	EPEC	Sampling time
TEER (Ohm∗cm^2^)	327 ± 68^a^	359 ± 89^a^	35 ± 38^b^	135 ± 116^b^	10 h
Mannitol flux (nmol∗cm^−2^∗h^−1^)	11 ± 3.9^a^	11.4 ± 7.2^a^	86.7 ± 26.6^b^	108 ± 25.9^b^	
*n* TEER∣*n* Mannitol	8∣5	9∣6	9∣6	7∣5	

Bacterial strains: Caco-2 cells were incubated with *E. faecium* (Ecf), ETEC, or EPEC for 10 h (mean ± standard deviation, variance analysis, post hoc Scheffe test, *P* ≤ 0.05, different letters indicate significant differences between incubation groups); *n* = cell culture inserts.
